# Health facility-based malaria surveillance: The effects of age, area of residence and diagnostics on test positivity rates

**DOI:** 10.1186/1475-2875-11-229

**Published:** 2012-07-07

**Authors:** Damon Francis, Anne Gasasira, Ruth Kigozi, Simon Kigozi, Sussann Nasr, Moses R Kamya, Grant Dorsey

**Affiliations:** 1 Department of Medicine, University of California, San Francisco General Hospital, 1001 Potrero Ave. Bldg. 30, Rm. 408, San Francisco, CA, 94110, USA; 2 Uganda Malaria Surveillance Project, PO Box 7475, Mulago Hospital Complex, Kampala, Uganda; 3 Malaria Branch, Centers for Disease Control and Prevention, 1600 Clifton Rd., Atlanta, GA, 30333, USA; 4 Department of Medicine, Makerere University School of Medicine, PO Box 7475, Mulago Hospital Complex, Kampala, Uganda

## Abstract

**Background:**

The malaria test positivity rate (TPR) is increasingly used as an indicator of malaria morbidity because TPR is based on laboratory-confirmed cases and is simple to incorporate into existing surveillance systems. However, temporal trends in TPR may reflect changes in factors associated with malaria rather than true changes in malaria morbidity. This study examines the effects of age, area of residence and diagnostic test on TPR at two health facilities in regions of Uganda with differing malaria endemicity.

**Methods:**

The analysis included data from diagnostic blood smears performed at health facilities in Walukuba and Aduku between January 2009 and December 2010. The associations between age and time and between age and TPR were evaluated independently to determine the potential for age to confound temporal trends in TPR. Subsequently, differences between observed TPR and TPR adjusted for age were compared to determine if confounding was present. A similar analysis was performed for area of residence. Temporal trends in observed TPR were compared to trends in TPR expected using rapid diagnostic tests, which were modelled based upon sensitivity and specificity in prior studies.

**Results:**

Age was independently associated with both TPR and time at both sites. At Aduku, age-adjusted TPR increased relative to observed TPR due to the association between younger age and TPR and the gradual increase in age distribution. At Walukuba, there were no clear differences between observed and age-adjusted TPR. Area of residence was independently associated with both TPR and time at both sites, though there were no clear differences in temporal trends in area of residence-adjusted TPR and observed TPR at either site. Expected TPR with pLDH- and HRP-2-based rapid diagnostic tests (RDTs) was higher than observed TPR at all time points at both sites.

**Conclusions:**

Adjusting for potential confounders such as age and area of residence can ensure that temporal trends in TPR due to confounding are not mistakenly ascribed to true changes in malaria morbidity. The potentially large effect of diagnostic test on TPR can be accounted for by calculating and adjusting for the sensitivity and specificity of the test used.

## Background

As malaria control efforts intensify, there is a vital need to accurately measure changes in the burden of disease and to evaluate the impact of control interventions through improved surveillance [1]. Malaria incidence, defined as the number of malaria cases per person-time, is a core indicator of the burden of disease and endorsed by the World Health Organization (WHO) [2]. For countries in sub-Saharan Africa where malaria morbidity is highest, malaria incidence is typically estimated based on the number of reported cases of malaria captured through the health management information system (HMIS) per population at risk. Incomplete reporting and lack of laboratory confirmation limit the accuracy of these data [2]. To account for those limitations, many countries also report the incidence of laboratory-confirmed cases, but these data may reflect the availability and utilization of clinical and laboratory services rather than malaria morbidity in the population [2]. Many studies of malaria control interventions such as indoor residual spraying or distribution of insecticide-treated nets use outcomes that are simpler to measure than malaria incidence, such as number of episodes of uncomplicated malaria (without a denominator), asymptomatic parasitaemia prevalence, haemoglobin levels, or all-cause child mortality [3,4].

The malaria test positivity rate (TPR), defined as the proportion of diagnostic tests that are positive for malaria, is an alternate indicator of malaria morbidity [2]. TPR is similar to the slide positivity rate (SPR) except that it includes the results of rapid diagnostic tests (RDTs) when they are used in addition to or in place of blood smears. TPR has been used as a surveillance indicator at the national [2] and regional level [5], and decreases in TPR have been used as evidence to support the effectiveness of malaria control interventions [6-8]. The advantages of TPR are that it inherently incorporates only laboratory-confirmed cases, provides a clear denominator and can provide a rapid and inexpensive means of assessing malaria morbidity in a population utilizing health care facilities where diagnostic testing is available. As it has been previously reported, a disadvantage of TPR is that differences over time or across populations may reflect differences in the incidence of non-malarial febrile illnesses rather than differences in the burden of malaria [9]. In addition, temporal trends in TPR may reflect changes in other factors associated with malaria diagnosis, such as the age or area of residence, the proportion and selection of individuals tested or the sensitivity and specificity of the diagnostic test used.

As the WHO now recommends laboratory confirmation for all patients suspected of having malaria before treating [10], the TPR has become an increasingly practical indicator of malaria morbidity. In this study, data from a health facility-based malaria surveillance system at two sites in Uganda with differing epidemiology were used to characterize the effects of age and area of residence on temporal trends in TPR. Temporal trends in TPR were also modelled using different diagnostic tests, including microscopy and RDTs, which would be expected affect TPR due to differences in sensitivity and specificity compared to microscopy.

## Methods

### Description of study sites and data collection

The Uganda Malaria Surveillance Project (UMSP) in collaboration with the Uganda National Malaria Control Programme (NMCP) established a health facility-based malaria surveillance system at six sentinel sites between September 2006 and January 2007. Detailed descriptions of study sites and data collection have been published previously [11]. Briefly, all sentinel site facilities are level IV government run health centres with a catchment population of approximately 100,000 people. They provide care free of charge, including diagnostic testing and medications. The two sites selected for this study, Aduku and Walukuba, represent contrasting malaria transmission settings in Uganda with previously reported entomological inoculation rates (infective mosquito bites per person per year) of 1,564 and 6, respectively [12]. This analysis included data collected between January 2009 and December 2010 at two of the six sentinel sites. Individual-level patient data collected for all patients presenting to the outpatient clinics of the health facilities included age, village and parish of residence, whether a blood smear was performed, presence or absence of asexual parasites based on a thick blood smear, in addition to other demographic information, basic clinical information, laboratory results, diagnoses, and treatments prescribed. Data were entered electronically using Epi Info version 3.5.1 (Centers for Disease Control and Prevention, Atlanta, GA, USA) at the sites and sent once a month to a core facility in Kampala for uploading to a SQL server (Microsoft Corporation, Redmond, WA, USA). A public website exists where standardized tables and figures can be generated to monitor trends in key indicators and monthly reports are posted [13].

### Definition of variables

Age data were collected as months and years up to age 5, and as years only after age five. Age was classified as less than 5, 5 to 15, and greater than 15 years in analyses of the relationships between age and TPR and between age and calendar time. In adjusting for age, visits at each site were separately categorized into 20 equivalent age quantiles, each containing 5% of visits at that site. Area of residence categories were determined based upon the reported parish of residence. Parishes that contributed to < 1% of cases that underwent diagnostic testing were combined into an “other” category. There were 24 categories of calendar time based upon the month and year. Suspected malaria was defined as all patients referred for malaria laboratory testing plus all patients not referred for a malaria laboratory test, but given a clinical diagnosis of malaria. TPR was defined as the proportion of tests (all of which were blood smears) positive for malaria.

Expected TPR (TPR_exp_) for an RDT was calculated from the observed TPR (TPR_obs_) as follows:

(1) $$TPR_{\exp}=(TPR_{\mathrm{obs}}\;x\;sensitivity)+(1-TPR_{\mathrm{obs}})\;x\;(1-specificity)$$

The sensitivity and specificity of RDTs was determined during a prior study at these sites conducted between May 2006 and February 2007 [14]. For histidine rich protein-2 (HRP-2) based RDTs (Paracheck; Orchid Biomedical Systems) sensitivity and specificity were 99.7% and 38.1% at Aduku, and 97.4% and 69.6% at Walukuba, respectively. Sensitivity and specificity of Plasmodium lactate dehydrogenase (pLDH) based RDTs (Parabank; Zephyr Biomedicals) were 98.6% and 69.9% at Aduku, and 92.3% and 81.9% at Walukuba, respectively.

### Statistical analysis

The potential for confounding by age and/or area of residence in the association between the exposure of interest, calendar time, and the outcome of interest, TPR, was investigated. Analyses of the relationships between age and area of residence and temporal trends in TPR included only those patients for whom a thick blood smear was performed.

To investigate the potential for age to confound the relationship between calendar time and TPR, the associations between age and calendar time and between age and TPR were evaluated separately using the Pearson Chi-square test. To visually inspect the degree to which confounding by age occurred, temporal trends in TPR were adjusted using direct standardization based on the distribution of visits among 20 age categories of equal size over the entire time period, and then compared to unadjusted temporal trends in TPR.

To investigate the potential for area of residence to confound the relationship between calendar time and TPR, the associations between area of residence and calendar time and between area of residence and TPR were also separately evaluated using the Pearson Chi-square test. To visually inspect the degree to which confounding by area of residence occurred, temporal trends in TPR were similarly adjusted for area of residence using direct standardization based on the distribution among parishes over the entire study period, and then compared to unadjusted temporal trends in TPR.

To characterize the effect of diagnostic test on temporal trends in TPR, an expected value for TPR was calculated using RDTs based on the sensitivity and specificity of those tests from a previous study from the same two sentinel sites using quality-controlled microscopy as a gold standard [14], as described above. All analyses were performed using R, version 2.9.1. P values <0.05 were considered statistically significant.

## Results

### Characteristics of visits

The characteristics of outpatient visits at the two sites are summarized in Table 1. More than 96% of visits had data on age and area of residence. The proportion of patients suspected of having malaria ranged from 53-55% at the two sites. At both sites, 95% of those with suspected malaria received a thick blood smear. A higher proportion of those receiving a thick blood smear were under five at Aduku compared to Walukuba (44% *vs* 29%, p < 0.001). Overall TPR for the entire study period was higher at Aduku compared to Walukuba (53% *vs* 40%, p < 0.001).

**Table 1:** Characteristics of outpatient visits at surveillance sites in 2009-10

**Characteristics**	**Surveillance Site**	
	**Walukuba**	**Aduku**
Visits with complete data* (% of total visits)	71,703 (98%)	38,912 (96%)
Number with suspected malaria (% with complete data)	37,806 (53%)	21,570 (55%)
Number with blood smear (% of suspected)	36,079 (95%)	20,488 (95%)
Under 5 (% of blood smears)	10,636 (29%)	9,052 (44%)
5 to 15 (% of blood smears)	8,755 (24%)	2,644 (13%)
Over 15 (% of blood smears)	16,688 (46%)	8,752 (43%)
Number with positive blood smear (TPR)	14,391 (40%)	10,806 (53%)

* Includes age and area of residence (parish).

### The effect of age on temporal trends in malaria test positivity rate

Changes in TPR over time could be confounded by age, which has a well known association with malaria infection and morbidity. As expected, TPR varied significantly by age group in Aduku. For visits by patients under five years of age, five to 15 and over 15, TPR was 71%, 64%, and 30%, respectively (p < 0.001). In Walukuba, the association between TPR and age was less dramatic (44%, 47%, and 34% for under five, five to 15 and over 15, respectively), but remained statistically significant (p < 0.001).

For age to confound temporal trends in malaria TPR, it must also be associated with calendar time, which was the case at both sites in this study. In Aduku, the proportion of visits by patients under age five varied from a high of 56% in September 2009 to a low of 24% in December 2010 (p < 0.001). There was an upward trend in age at Aduku throughout the study period with the proportion of visits by patients under age five ranging between 44% and 55% in the first six months of the study period, and between 24% and 40% in the final six months. Walukuba demonstrated less variation in the distribution of age over time, and did not exhibit any consistent trend, but differences remained statistically significant. The proportion of patients under age five varied from a high of 41% in April 2009 to a low of 23% in June 2010 (p < 0.001).

**Figure 1:**
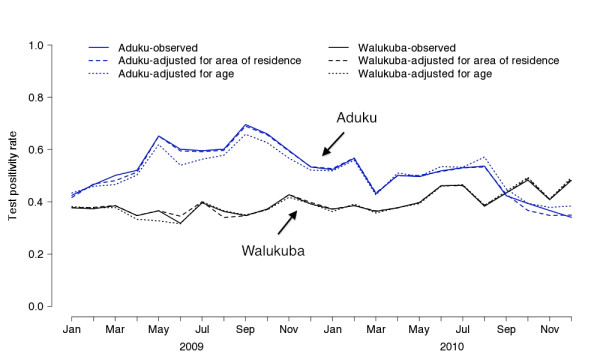
TPR-observed, TPR-adjusted for age, and TPR-adjusted for area of residence at Walukuba and Aduku health centers (TPR = test positivity rate).

Given the significant associations between both age and calendar time, and age and TPR, the potential for confounding was present at both sites. To determine the degree to which confounding actually occurred, temporal trends in the observed TPR and TPR adjusted for age were compared as shown in Figure1. Subtle but clear confounding by age was observed in Aduku, where the difference in TPR adjusted for age compared to the observed TPR gradually increased over calendar time. This bias could be predicted based upon the gradual increase in age, and the strong association between young age and a positive thick blood smear. As a result of confounding by age, the decline in the observed TPR from 69% in September 2009 to 34% in December 2010, was greater than the decline in TPR adjusted for age from 66% to 38% over the same time period. In contrast, Walukuba demonstrates minimal evidence of confounding by age as the temporal trends in observed TPR and TPR adjusted for age are nearly identical over the entire study period. The largest difference in the monthly trend occurs between May and June of 2009 when the observed TPR declined 5% and the TPR adjusted for age declined 1%.

### The effect of area of residence on trends in malaria test positivity rate

The association between area of residence and TPR is also well known, and was of interest in this study. Visits were categorized based on the parish where the patient lived, and visits from parishes contributing fewer than 1% of the cases for the entire study period were grouped into a category labelled “Other”. This created 8 regions surrounding Walukuba and 18 surrounding Aduku. Table 2 shows the frequency distribution of the area of residence categories, average distance from the parish to the health facility and TPR over the entire study period in the regions surrounding Walukuba and Aduku. In Walukuba, TPR varied significantly across the areas of residence (p < 0.001), ranging from 38% to 53%. In Aduku, TPR varied significantly across the areas of residence (p < 0.001), ranging from 41% to 60%. There was no clear pattern between the distance from the area of residence to the clinic and the TPR at either site.

**Table 2:** Distribution of area of residence and TPR

**Surveillance Site**							
**Walukuba**				**Aduku**			
**Parish**	Distance*	**Frequency**	**TPR**	**Parish**	Distance*	**Frequency**	**TPR**
Masese	3.8	33.9%	4639/12212 (38.0%)	Ongoceng	3.5	20.6%	2271/4205 (54.0%)
Walukuba West	0.7	27.3%	3908/9843 (39.7%)	Aboko	5.5	15.1%	1838/3089 (59.5%)
Walukuba East	0.5	20.9%	2917/7535 (38.7%)	Adyeda	5.7	13.7%	1464/2797 (52.3%)
Bugembe	4.2	4.4%	708/1590 (44.5%)	Apire	6.2	8.4%	933/1712 (54.5%)
Mpumudde	3.6	2.0%	303/727 (41.7%)	Alira	8.6	8.0%	880/1632 (53.9%)
Mafubira	4.7	1.6%	270/586 (46.1%)	Abany	6.6	7.5%	859/1535 (56.0%)
Central Jinja East	1.9	1.1%	206/388 (53.1%)	Atongtidi	12.4	3.6%	361/726 (49.7%)
Others**	N/A	8.9%	1440/3198 (45.0%)	Anwangi	11.0	2.8%	280/578 (48.4%)
				Inomo	10.4	2.7%	269/553 (48.6%)
				Abedmot	13.3	1.7%	150/353 (42.5%)
				Agwiciri	13.0	1.6%	179/316 (56.7%)
				Akali	12.9	1.4%	144/290 (50.0%)
				Aornga	14.0	1.4%	129/289 (44.6%)
				Ajok	16.4	1.4%	116/280 (41.4%)
				Acaba	15.2	1.1%	104/224 (46.4%)
				Abedi	14.3	1.1%	111/222 (50.0%)
				Etekober	15.2	1.1%	100/216 (46.3%)
				Others**	N/A	7.0%	618/1431 (43.2%)
**Total**	**N/A**	**100%**	**14391/36079 (39.9%)**	**Total**	**N/A**	**100%**	**10806/20448 (52.9%)**

* Distance from center of parish to sentinel site health facility in km.

** Combinations of all parishes with frequencies < 1%.

Although there were significant associations between area of residence and TPR at both sites, the distribution of area of residence among patients undergoing diagnostic testing would need to vary significantly over time for the potential for confounding to be present, and this was the case at both sites. For example, the proportion of patients who underwent diagnostic testing who were from the Masese Parish ranged from 30% in October 2009 to 39% in December 2010 at Walukuba and patients who were from the Ongoceng Parish ranged from 17% in August 2009 to 25% in October 2009 at Aduku (p < 0.001 in both cases).

Given the associations with both TPR and calendar time, area of residence was a potential confounder. As was done for age, temporal trends in observed TPR and TPR adjusted for area of residence were compared, however, they differed only slightly and without any clear pattern (Figure1). The largest difference in monthly trends at Aduku occurred between November 2010 and December 2010 when observed TPR declined 3% and TPR adjusted for area of residence was unchanged. In Walukuba, the largest difference in monthly trends occurred between May and June of 2009 when observed TPR decreased 5% and TPR adjusted for area of residence decreased 2%.

### The effect of diagnostic test on trends in malaria test positivity rate

**Figure 2:**
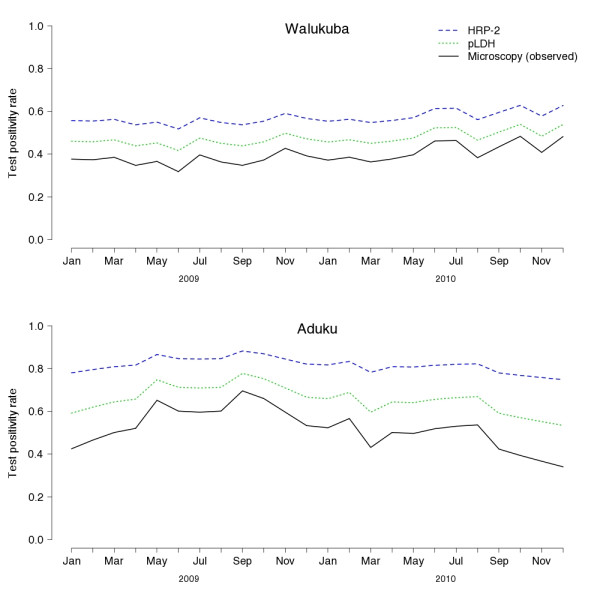
TPR with microscopy (observed) and expected TPR with pLDH- and HRP-2-based RDTs (TPR = test positivity rate).

Figure2 shows trends in observed TPR using microscopy compared to those expected using RDTs based on HRP-2 and pLDH. At both sites, expected TPR with pLDH- and HRP-2-based RDTs is higher than observed TPR. The differences are greater at Aduku than at Walukuba, and greater with HRP-2-based RDTs than with pLDH-based RDTs. All trends for observed and expected TPR move in the same direction each month over the entire study period. However, it is notable that the expected trends in TPR with RDTs are flatter than trends in observed TPR, most obviously in the case of expected TPR with HRP-2-based RDTs at Aduku. For example, the highest and lowest values, which occurred in September 2009 and December 2010, respectively, were 69% and 34% for the observed TPR and 88% and 75% for the expected TPR with the HRP-2-based RDT.

## Discussion

TPR is increasingly used as an indicator of temporal trends in malaria morbidity. Ideally, changes in TPR over time will reflect true changes in malaria incidence for a population of interest. However, several factors including potential confounders such as age and area of residence, proportion of cases subjected to testing, care-seeking and utilization trends, and choice of diagnostic test may cause changes in TPR independent of true changes in the incidence of malaria. In this study, the effects of age, area of residence, and diagnostic test on TPR were investigated at two sites with different transmission intensity in Uganda. Age and area of residence demonstrated the potential to be important confounders at both sites given their independent associations with both time and TPR. However, controlling for each of them had only a small effect on the trends in TPR at the two sites. The directions of trends in the expected TPR using pLDH- and HRP-2-based RDTs were similar to trends in observed TPR, but there were differences between the values of observed and expected TPR at all time points. These differences were more pronounced at Aduku, the higher transmission site, and more pronounced using the HRP-2-based RDT.

Potential confounders are an important consideration in observational studies assessing any association, including temporal trends, which represent associations between time and an indicator of interest, in this case TPR. Any factor associated with both the exposure of interest (calendar time) and the outcome of interest (TPR) has the potential to confound temporal trends in TPR. Numerous factors may be associated with both time and malaria incidence such as weather, precipitation patterns, proportion of patients tested, care-seeking and utilization and home construction. Age and area of residence were chosen for analysis because they are well known to be associated with malaria incidence, and they can both be easily measured.

In the case of age, the expected association between younger age and higher TPR was observed. The age distribution of patients also differed significantly by calendar time, particularly at Aduku where the population receiving a malaria blood smear became gradually older over time. The comparison of temporal trends in observed TPR and age-adjusted TPR in Aduku provides a subtle demonstration of confounding in which age-adjusted TPR increased over time relative to the observed TPR due to the gradual increase in age over time and the lower TPR in older patients. Although confounding by age only had a modest effect on temporal trends in TPR in this study, the effect could be larger in other circumstances, for example a large increase in paediatric capacity at the clinic where surveillance is being conducted.

TPR was also associated with area of residence, though there were no clear patterns relative to distance from the clinic. In the case of Aduku, the TPR was lower outside the catchment area compared to near the clinic, whereas the opposite was true in Walukuba. As with the age distribution, the distribution of area of residence for patients receiving a blood smear also varied over time at both sites. However, there was no noticeable confounding of temporal trends in TPR by area of residence at either site. Nonetheless, it is easy to imagine circumstances in which confounding of temporal trends in TPR by area of residence may be important such as changes in the availability of transportation to the clinic from one area relative to another with a substantially different malaria burden. Controlling for factors such as age and area of residence with methods such as direct standardization or stratification can assure that changes in TPR over time that are due to confounding by these factors are not mistakenly ascribed to changes in malaria morbidity.

The choice of diagnostic test can also affect the interpretation of trends in TPR in two important ways. First, even when the proportion of patients with true infections stays the same, a change from one diagnostic test to another could cause a change in TPR that is exclusively due to a change in the proportion of true positive and false positive tests. Separately reporting the TPR for microscopy and RDTs, as is done in the World Malaria Report [2], partially addresses this problem. However, it still would not account for a substantial change in the quality of microscopy, which can be widely variable [15,16], or a change from one RDT to another with different sensitivity and specificity [14]. Second, the choice of diagnostic test affects the slope of the trends in TPR, and therefore the ability to distinguish a real difference in malaria morbidity. A low specificity test, which generates more false positives, will tend to obscure trends within the upper range of TPR values (closer to 1), whereas a low sensitivity test, which generates more false negatives, will obscure trends within the lower range of TPR values (closer to 0). High transmission sites such as Aduku are more likely to have a higher TPR, and are more likely to suffer from decreased specificity of RDTs, presumably due to frequent infections and persistence of parasite antigens after resolution of infection [14]. This effect is demonstrated in the comparison between a relatively large decrease in observed TPR at Aduku between September 2009 and December 2010 and a much smaller decrease in the expected TPR with an HRP-2-based RDT (Figure2). These two effects of diagnostic test on trends in TPR can be accounted for by calculating the sensitivity and specificity of diagnostic tests periodically *via* comparison with a gold standard at the health facilities of interest. Using those results, the TPR can be adjusted accordingly based on the equation shown earlier.

This study has several important limitations. First, the sensitivity and specificity of RDTs at these sites using microscopy as a gold standard may have changed between the time of the study referenced for those values [14] and this study. Sensitivity and specificity of RDTs have been reported to vary based upon clinical and epidemiologic setting, most often related to differences in the distribution of parasite densities among infected patients, and based upon changes in storage and usage of the tests [17]. Such a change may have affected the magnitude of differences between the values and slopes of observed and expected TPR, but the general direction of those differences likely would have been the same. Second, given the large samples sizes in this study, it is not surprising that statistically significant differences were found between potential confounders and the exposure (calendar time) and outcome (TPR) of interest. Indeed, the magnitude of these differences were of questionable relevance in terms of potential confounding and temporal trends in TPR based on adjusted analyses did not reveal differences compared to the unadjusted temporal trends that would likely be of public health importance. Third, this study was limited to two sentinel surveillance sites in Uganda, and may not be representative of many areas of the world with lower transmission intensity. TPR is not very useful as a surveillance indicator in settings with very low transmission intensity, though a TPR below 5% has been recommended as one of the criteria for readiness to shift to the elimination phase of malaria control [2]. Finally, even in settings with malaria transmission on the order of that in these two study sites, TPR has many important limitations as an indicator of malaria burden which have been discussed elsewhere - the numerical change in TPR does not reflect either linear or proportional changes in malaria incidence in the population sampled, it is useful to estimate relative changes in malaria incidence but it cannot be used to estimate the actual incidence or compare incidence across sites, and changes in its value could be caused by changes in non-malarial fevers, the population of patients accessing the health facility, or changes in testing practices at the health facility [9].

## Conclusions

TPR is a key malaria surveillance indicator in resource-limited settings with medium to high transmission. It is easily integrated into HMIS reporting, and its reliability will depend less upon stable clinic attendance as do estimates of malaria incidence based on clinical or laboratory diagnosis, so long as a consistently high and representative proportion of clients access health facilities and are offered a diagnostic test. A thorough understanding of both the limitations of TPR and methods for improving its accuracy is important for monitoring the effectiveness of malaria control interventions. Indeed, improved methods to quantify and compare changes in TPR in different settings could be used to provide more practice-based evidence on the relative effectiveness of malaria control interventions. The urgency of the overall burden of malaria, the increasing availability of new tools to fight malaria, and the tremendous resources required for controlled experiments of public health interventions demand creative techniques such as health facility-based surveillance with indicators like TPR to efficiently test, refine, and deploy the next generation of strategies for malaria control.

## Competing interests

The authors declare that they have no competing interests.

## Authors’ contributions

AG, RK, SPK, SN, MRK and GD contributed to study design and oversight. DF, AG, and GD contributed to methodology, data analysis, interpretation of results, and drafting of the manuscript. All authors read and approved the final manuscript.
